# Bacterial Indole as a Multifunctional Regulator of Klebsiella oxytoca Complex Enterotoxicity

**DOI:** 10.1128/mbio.03752-21

**Published:** 2022-01-25

**Authors:** Nagender Ledala, Mishika Malik, Karim Rezaul, Sara Paveglio, Anthony Provatas, Aaron Kiel, Melissa Caimano, Yanjiao Zhou, Jonathan Lindgren, Kristyna Krasulova, Peter Illes, Zdeněk Dvořák, Sandhya Kortagere, Sabine Kienesberger, Amar Cosic, Lisa Pöltl, Ellen L. Zechner, Subho Ghosh, Sridhar Mani, Justin D. Radolf, Adam P. Matson

**Affiliations:** a Department of Pediatrics, UConn Health, Farmington, Connecticut, USA; b Division of Neonatology, Connecticut Children’s Medical Center, Hartford, Connecticut, USA; c Department of Immunology, UConn Health, Farmington, Connecticut, USA; d Department of Medicine, UConn Health, Farmington, Connecticut, USA; e Department of Genetics and Genome Sciences, UConn Health, Farmington, Connecticut, USA; f The Jackson Laboratory for Genomic Medicine, Farmington, Connecticut, USA; g Center for Environmental Sciences and Engineering, University of Connecticut, Storrs, Connecticut, USA; h Department of Molecular Biology and Biophysics, UConn Health, Farmington, Connecticut, USA; i Department of Cell Biology and Genetics, Faculty of Science, Palacky University, Olomouc, Czech Republic; j Department of Microbiology & Immunology, Drexel University, Philadelphia, Pennsylvania, USA; k Institute of Molecular Biosciences, University of Graz, BioTechMed-Graz, Graz, Austria; l Department of Medicine, Molecular Pharmacology and Genetics, Albert Einstein College of Medicine, Bronx, New York, USA; University of Oklahoma Health Sciences Center

**Keywords:** *Klebsiella oxytoca* complex, pregnane X receptor, cytotoxin, indole, intestinal inflammation

## Abstract

Gastrointestinal microbes respond to biochemical metabolites that coordinate their behaviors. Here, we demonstrate that bacterial indole functions as a multifactorial mitigator of Klebsiella grimontii and Klebsiella oxytoca pathogenicity. These closely related microbes produce the enterotoxins tilimycin and tilivalline; cytotoxin-producing strains are the causative agent of antibiotic-associated hemorrhagic colitis and have been associated with necrotizing enterocolitis of premature infants. We demonstrate that carbohydrates induce cytotoxin synthesis while concurrently repressing indole biosynthesis. Conversely, indole represses cytotoxin production. In both cases, the alterations stemmed from differential transcription of *npsA* and *npsB*, key genes involved in tilimycin biosynthesis. Indole also enhances conversion of tilimycin to tilivalline, an indole analog with reduced cytotoxicity. In this context, we established that tilivalline, but not tilimycin, is a strong agonist of pregnane X receptor (PXR), a master regulator of xenobiotic detoxification and intestinal inflammation. Tilivalline binding upregulated PXR-responsive detoxifying genes and inhibited tubulin-directed toxicity. Bacterial indole, therefore, acts in a multifunctional manner to mitigate cytotoxicity by Klebsiella spp.: suppression of toxin production, enhanced conversion of tilimycin to tilivalline, and activation of PXR.

## INTRODUCTION

The human gastrointestinal tract represents a complex ecosystem comprised of resident microbiota, dietary elements, and host factors. Within this environment, microbe-microbe and host-microbe interactions are important for maintaining homeostasis (1, 2). Alterations in the gut ecosystem by factors such as diet, antibiotics, and host susceptibility can lead to dysbiotic conditions wherein a resident microbe adopts pathogenic behaviors and causes disease (3, 4).

Indole is a microbiota-derived signaling molecule present in the human gut that is known to modulate virulence factors in several enteric bacteria (5–9) while concurrently functioning to strengthen the host intestinal barrier (10). Indole and indole derivatives also activate intestinal xenobiotic receptors, such as pregnane X receptor (PXR) (11), to facilitate anti-inflammatory (12) and detoxification (13) responses. The levels of indole in the intestinal lumen are unknown; however, concentrations in human feces can reach 6 mM (14), with most studies describing ranges between 0.25 and 1.1 mM (15, 16). Indole is generated as a by-product of the reversible conversion of l-tryptophan to pyruvate, a key glycolytic end product and Krebs cycle intermediate, by the enzyme tryptophanase (17). The gene encoding tryptophanase, *tnaA*, is ubiquitous among gut bacteria (18). In Escherichia coli, the transcription of *tnaA* is regulated by carbon catabolite repression; the availability of a preferred carbon source, such as glucose, eliminates the need to convert tryptophan into pyruvate and indole, which is subsequently released (19). While it is unclear if other gut bacteria regulate indole production in the same way, the abundance and metabolic status of tryptophanase producers could profoundly influence luminal indole levels.

Klebsiella oxytoca is regarded as a human gut commensal, yet in older children and adults treated with β-lactam antibiotics, overgrowth of cytotoxin-producing strains of K. oxytoca results in antibiotic-associated hemorrhagic colitis (AAHC), a distinct form of non-Clostridium difficile colitis (20, 21). Recently, we found that blooms of cytotoxin-producing K. oxytoca also were associated with the development of necrotizing enterocolitis (NEC) (22), a devastating intestinal disease of premature infants (23). Similar strains were found at lower abundance in the majority of non-NEC control infants (22), which underscores the importance of deciphering how contextual changes in the gut ecosystem impact pathogenicity. In heathy adults, intestinal colonization with K. oxytoca is reported in 2% to 9% of subjects and ∼1/2 of the strains produce cytotoxin (24). Recent studies indicate more prevalent carriage of this microbe in the neonatal population (25) and that many isolates from infants have the capacity to produce toxin (22, 26).

K. oxytoca (*sensu stricto*) is a member of the K. oxytoca complex, comprising several closely related species within different phylogroups named according to the presence of a *bla*_OXY_ variant (β-lactamase) conferring resistance to amino- and carboxypenicillin (27). Methods used in clinical microbiology laboratories often identify all K. oxytoca complex isolates as K. oxytoca (*sensu lato*). The identity and contributions of several members to disease remained largely unknown until whole-genome sequencing (WGS) and other advancements led to the identification of newer strains and an expansion of the K. oxytoca complex (28–32). Klebsiella grimontii and other K. oxytoca complex members subsequently were demonstrated to inhabit the gut of premature infants (33, 34).

Identification of a biosynthetic gene cluster responsible for generating the pyrrolobenzodiazepine cytotoxins tilimycin and tilivalline has enabled genetic screening for cytotoxin-producing members of the K. oxytoca complex (22, 26, 34, 35). Analysis of this gene cluster in the K. oxytoca AAHC isolate AHC-6 showed that products of *npsA* and *npsB* are involved in the late-step enzymatic synthesis of tilimycin (also called kleboxymycin), a toxic product of nonribosomal peptide synthesis (NRPS) (36, 37). In the presence of indole, tilimycin spontaneously incorporates the indole ring to form tilivalline (36). While both tilimycin and tilivalline are considered toxins, they have distinct molecular targets, and tilimycin is substantially more cytotoxic to mammalian cells than tilivalline (38). Host factors also are involved in determining sensitivity to exogenous compounds. PXR, a ligand-activated host nuclear receptor, plays an important role in xenobiotic detoxification (13). PXR also regulates intestinal inflammatory responses through the binding of microbe-specific indoles (12).

Here, we demonstrate that bacterial indole alleviates cytotoxicity induced by *K. grimontii* and K. oxytoca via suppression of tilimycin synthesis and enhanced conversion of tilimycin to tilivalline while simultaneously activating a host nuclear receptor, PXR. We establish that tilivalline is a strong agonist of PXR; this interaction upregulates PXR-responsive detoxifying genes and mitigates tubulin acetylation. Our findings strengthen the notion that metabolites produced by gut microbes are critical mediators of microbe-microbe and host-microbe cross talk.

## RESULTS

### Taxonomic reclassification of UCH-1 as *K. grimontii*.

Our previous study with isolates from NEC stool samples identified cytotoxin-producing K. oxytoca organisms belonging to different sequence types (22). In light of the recent identification of novel species in the K. oxytoca complex (28–31), we conducted further taxonomic analysis of our NEC isolate UCH-1 (22). We compared the average nucleotide identity (ANI)/OrthoANI (39) of UCH-1 (BioProject accession number PRJNA608440; SRA accession number SAMN15690275) across various phylogroup members (see Table S1 in the supplemental material). All of the Ko phylogroups except Ko6 showed ANI values equal to or lower than the suggested bacterial species boundary ANI values of 96% or higher (40), leading us to classify UCH-1 as *K. grimontii*. To confirm this taxonomic assignment, we performed a phenol red d-melezitose fermentation test (29). UCH-1 growing in phenol red melezitose broth showed no color change compared to the K. oxytoca reference strain ATCC 13182, whereas a positive color change (yellow) was observed for both UCH-1 and K. oxytoca ATCC 13182 cultured in phenol red glucose broth (Fig. S1).

10.1128/mbio.03752-21.1 FIG S1 Carbohydrate fermentation tests to confirm the taxonomic assignment of UCH-1 as *K. grimontii*. Phenol red broth tubes containing d-melezitose (PRM) or glucose (PRG) were inoculated with ATCC 13182 (K. oxytoca) or UCH-1. Tubes containing UCH-1 growing in PRM showed no color change compared to those containing K. oxytoca, whereas a positive color change (yellow) was observed for both UCH-1 and K. oxytoca cultured in PRG. Download FIG S1, TIF file, 0.5 MB. Copyright © 2022 Ledala et al. 2022 Ledala et al. https://creativecommons.org/licenses/by/4.0/ This content is distributed under the terms of the Creative Commons Attribution 4.0 International license.

10.1128/mbio.03752-21.7 TABLE S1 Taxonomic classification of UCH-1. Download Table S1, XLSX file, 0.01 MB. Copyright © 2022 Ledala et al. 2022 Ledala et al. https://creativecommons.org/licenses/by/4.0/ This content is distributed under the terms of the Creative Commons Attribution 4.0 International license.

### Glucose differentially impacts tilimycin and indole biosynthesis by *K. grimontii* strain UCH-1.

**FIG 1:**
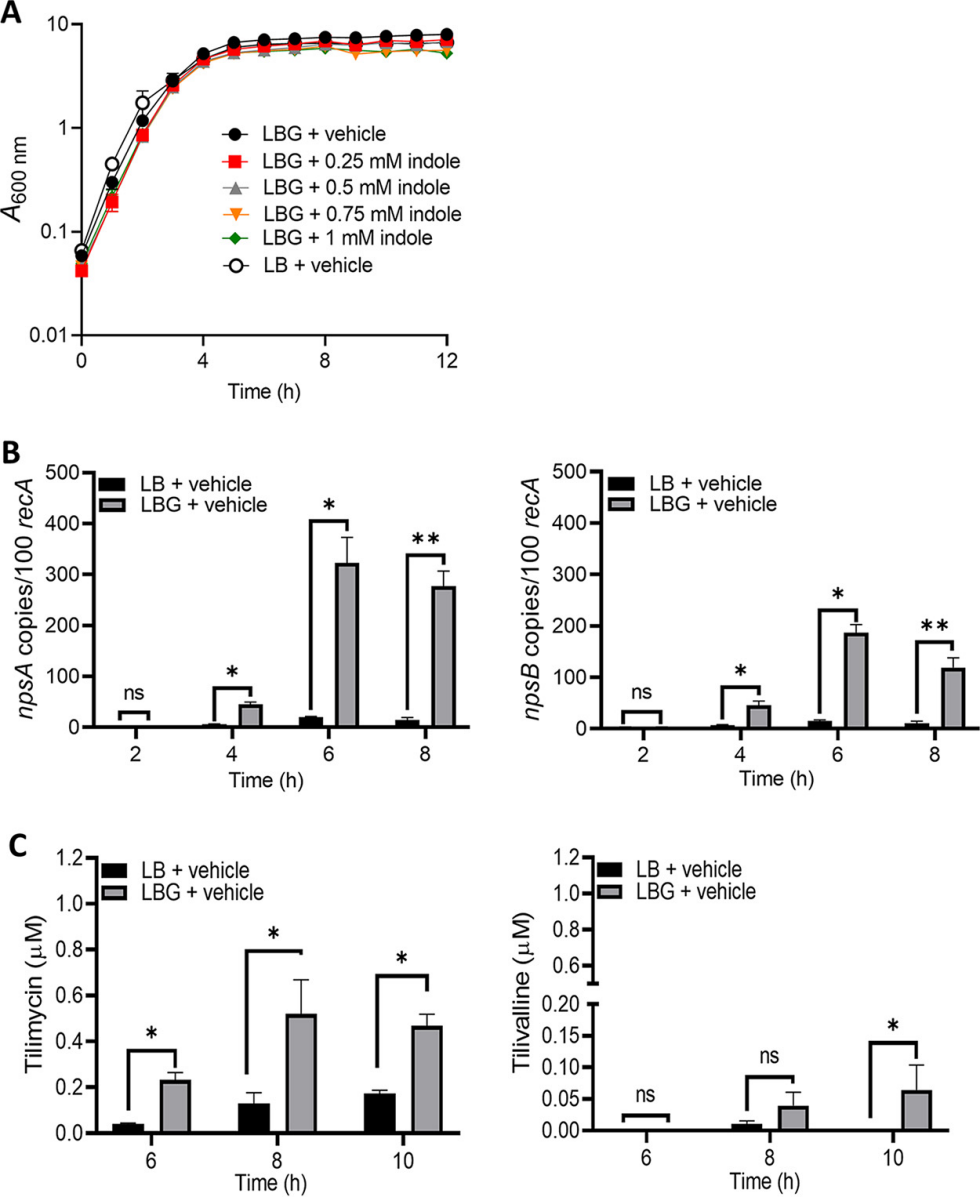
Glucose induces cytotoxin synthesis by UCH-1. (A) UCH-1 was grown in LB broth alone or with glucose (LBG) containing indole at various concentrations with shaking at 37°C for 12 h (*n *= 3 to 8). (B) Effect of glucose on *npsA* and *npsB* expression. Transcript copy numbers of *npsA* and *npsB* (per 100 copies of *recA* transcript) in samples obtained at exponential (2 to 4 h) and postexponential (6 to 8 h) growth phases were determined by qRT-PCR (*n *= 3 to 8). (C) Tilimycin and tilivalline levels in postexponential growth phase culture supernatants determined by LC-MS (*n *= 3). The data are presented as mean values ± standard errors of the means. Statistical analysis was done by Mann-Whitney U test. Where stated, “vehicle” means addition of DMF (0.1%). *, *P ≤ *0.05; **, *P ≤ *0.01. ns, not significant.

The addition of glucose or lactose to the growth medium is reported to substantially enhance the cytopathic effects of K. oxytoca culture supernatants (37). To understand the relationship between growth conditions and cytotoxin synthesis by *K. grimontii*, UCH-1 was cultured in Luria-Bertani (Lennox) broth (LB) without and with glucose at physiologically relevant luminal concentrations (2.5 mg/mL or 13.88 mM) (LBG) (41), and growth curves were generated. The addition of glucose to LB medium, which is otherwise devoid of carbohydrates, negligibly affected growth of UCH-1 (Fig. 1A). Expression levels of *npsA* and *npsB*, key genes involved in the tilimycin biosynthetic pathway, then were determined at 2, 4, 6, and 8 h, representing, respectively, early exponential, mid-exponential, and postexponential and stationary phases of growth. As shown in Fig. 1A and B, the addition of glucose resulted in a dramatic increase in expression of both *npsA* and *npsB*, with maximal expression occurring at 6 to 8 h, when UCH-1 entered postexponential growth phase. Consistent with the transcriptional data, mass spectrometry (MS) analysis of culture supernatants demonstrated that glucose increased synthesis of tilimycin and tilivalline, with 5- to 10-fold-greater concentrations of tilimycin than tilivalline (Fig. 1C). In control cultures without glucose, tilimycin concentrations were significantly lower, with little tilivalline detected (Fig. 1C).

**FIG 2:**
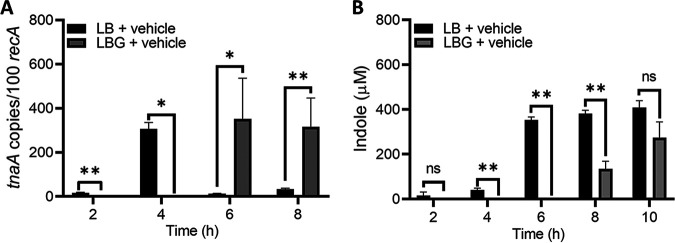
Glucose represses expression of *tnaA* and indole biosynthesis by UCH-1. (A) *tnaA* transcript copy numbers (per 100 copies of *recA* transcripts) in samples obtained at exponential (2 to 4 h) and postexponential (6 to 8 h) growth phases determined by qRT-PCR (*n *= 3 to 8). (B) Indole levels in exponential- to postexponential-growth-phase culture supernatants determined by LC-MS (*n *= 3 to 7). The data are presented as mean values ± standard errors of the means. Statistical analysis was done by Mann-Whitney *U* test.*, *P ≤ *0.05; **, *P ≤ *0.01.

Indole is essential for the nonenzymatic conversion of tilimycin to tilivalline (36). Since carbohydrates also are known to regulate expression of *tnaA* and indole synthesis in E. coli (19), we next investigated the effects of glucose on indole production by UCH-1. The isolate was cultured in LB broth without and with glucose as described above. The addition of glucose to the culture medium significantly repressed transcription of *tnaA* during early exponential and mid-exponential growth (2 to 4 h), whereas there was derepression thereafter (Fig. 2A). In cultures without glucose, expression of *tnaA* was observed as early as 2 h, with the highest expression at 4 h; at later time points, normalized transcript levels for *tnaA* were markedly reduced. MS analysis of culture supernatants demonstrated that in the absence of glucose, indole was readily produced at early time points (2 to 6 h), while the addition of glucose resulted in essentially no measurable indole (Fig. 2B). At later time points (8 to 10 h), indole was detected when UCH-1 was grown with and without glucose, possibly reflecting decreased carbohydrate availability in the medium with added glucose. Thus, expression of *tnaA* in *K. grimontii* appears to be controlled by catabolite repression.

### Exogenous indole inhibits K. oxytoca complex cytotoxin production.

**FIG 3:**
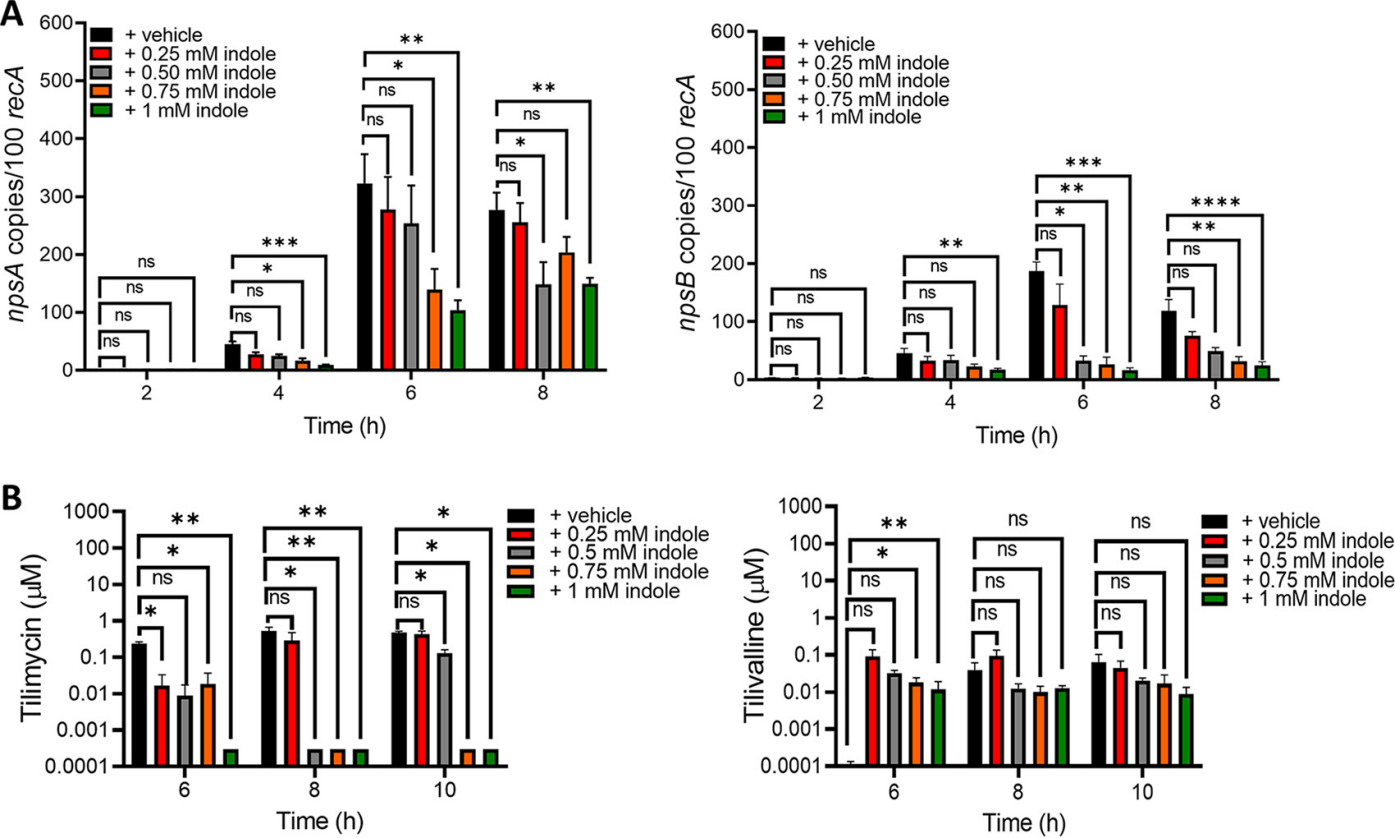
Exogenous indole represses cytotoxin synthesis by UCH-1. (A) Expression of the *npsA* and *npsB* genes in response to increasing concentrations of indole. Transcript copy numbers for *npsA* and *npsB* (per 100 copies of *recA* transcript) in samples obtained at exponential (2 to 4 h) and postexponential (6 to 8 h) growth phases were determined by qRT-PCR (*n *= 3 to 8). (B) Analysis of tilimycin and tilivalline metabolite levels in exponential- to postexponential-growth-phase culture supernatants by LC-MS (*n *= 3). The data are presented as the means values ± standard errors of the means. Statistical analysis was done by Kruskal-Wallis test followed by Dunn’s posttest. *, *P ≤ *0.05; **, *P ≤ *0.01; ***, *P ≤ *0.001; ****, *P ≤ *0.0001.

Perturbations in environmental indole concentrations alter expression of virulence factors in several gut pathogens (5–9). We next examined the effect of exogenous indole on the cytotoxic properties of *K. grimontii*. UCH-1 was cultured in LBG with and without increasing concentrations of indole, and growth curves were generated. The addition of indole in physiologic ranges of 0.25 to 1.00 mM (15, 16) negligibly affected growth (Fig. 1A); however, indole markedly repressed the expression of both *npsA* and *npsB* in a dose-dependent manner, with *npsB* demonstrating more marked repression during postexponential growth (Fig. 3A). Transcript levels for *npsB* at 0.50 mM to 1.0 mM indole concentrations were nearly 5- to 10-fold lower than those in control (vehicle) samples (Fig. 3A). Congruent with these transcriptional data, MS analysis of culture supernatants obtained at 6, 8, and 10 h demonstrated that exogenous indole dramatically reduced tilimycin concentrations below detection levels in a dose-dependent manner (Fig. 3B). Tilivalline concentrations remained low regardless of whether indole was added, indicating that indole-driven conversion of tilimycin to tilivalline was not a major factor in the reduction of tilimycin (Fig. 3B). Culture supernatants of UCH-1 in LBG plus a vehicle at 2 and 4 h demonstrated little tilimycin or tilivalline (tilimycin, 0.02 μM; tilivalline, not detected [ND]).

**FIG 4:**
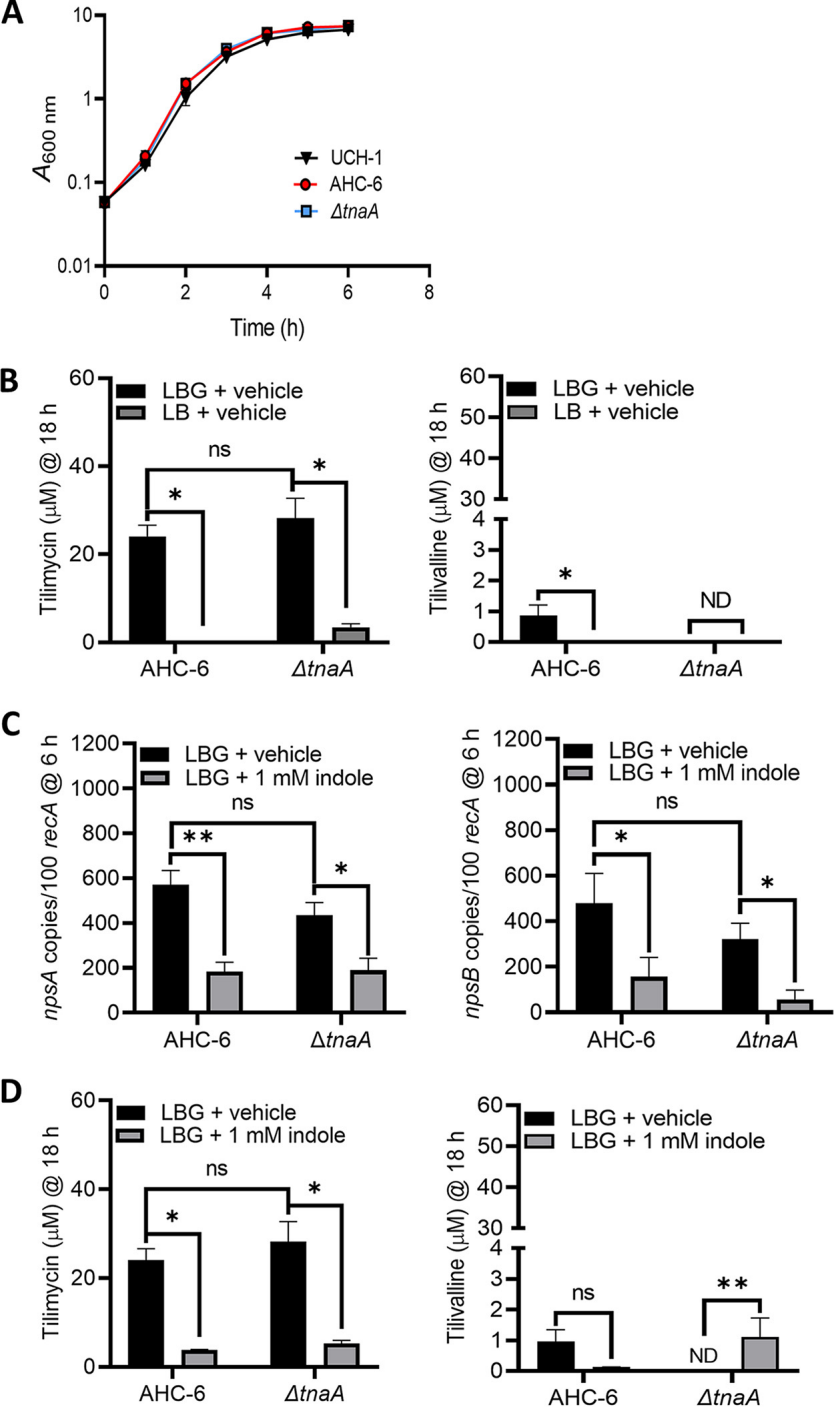
Glucose and indole reciprocally regulate cytotoxin synthesis by K. oxytoca strain AHC-6 and the AHC-6Δ*tnaA* mutant. (A) Comparison of UCH-1 growth with AHC-6 and AHC-6Δ*tnaA* in LB medium with glucose (LBG) at 37°C for 6 h (*n *= 3 to 7). (B) Analysis of tilimycin and tilivalline metabolite levels from 18-h culture supernatants of AHC-6 and AHC-6 Δ*tnaA* grown in LB medium with or without added glucose determined by LC-MS. ND, not detected (*n *= 3 to 8). (C) Effect of indole (1 mM) on *npsA* and *npsB* expression in AHC-6 and AHC-6 Δ*tnaA* grown in LBG. Transcript copy numbers for *npsA* and *npsB* (per 100 copies of *recA* transcript) in samples obtained at postexponential (6 h) growth phase (*n *= 6 or 7) were determined by RT-qPCR. (D) Effect of indole (1 mM) on tilimycin and tilivalline metabolite levels from 18-h culture supernatants of AHC-6 and AHC-6 Δ*tnaA* grown in LBG determined by LC-MS (*n *= 3 to 7). The data represent mean values ± standard errors of the means. Statistical analysis was done by Mann-Whitney U test. In samples where no tilivalline was detected, the detection limit by LC-MS was used for statistics. Where stated, “vehicle” means addition of DMF (0.1%). *, *P ≤ *0.05; **, *P ≤ *0.01.

**FIG 5:**
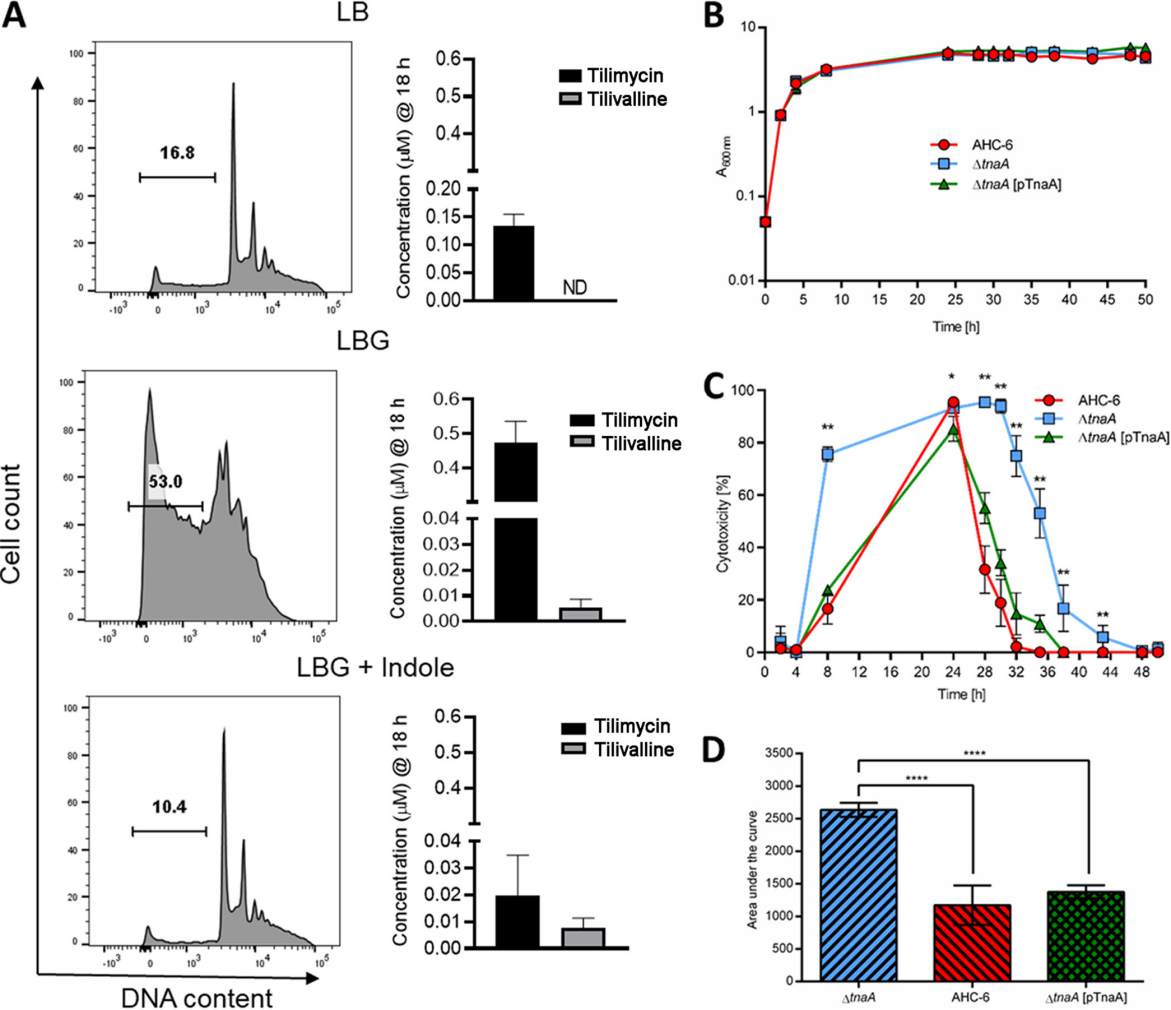
Enterotoxicity of K. oxytoca complex is differentially regulated by glucose and indole. (A) Filtered supernatants from *K. grimontii* strain UCH-1 cultures, grown for 18 h in LB medium, LBG, or LBG plus 1.0 mM indole, were applied to T84 enterocytes. Shown are percentages of sub-G1 apoptotic cells following 72 h of exposure as determined by flow cytometry. Concentrations of tilimycin and tilivalline in the culture supernatants were determined by LC-MS (*n *= 3 to 8). The data are representative images or mean values ± standard errors of the means. (B) Growth comparison of indole-producing K. oxytoca AHC-6, the indole-deficient AHC-6 Δ*tnaA*, and complementing strain AHC-6 Δ*tnaA* (pTnaA) grown in CASO medium at 37°C (*n *= 3). The data represent mean values ± SD. (C) Cytotoxicity of filtered supernatants collected from cells used for panel B on HeLa cells (*n* = 3; measured in triplicates). Shown are reciprocal values (means ± SD) of surviving HeLa cells after treatment with 1/27 dilutions of supernatants. Statistical comparison (Mann-Whitney) of AHC-6 and AHC-6 Δ*tnaA* was done for each time point. *, *P < *0.05; **, *P < *0.01. (D) Statistical analysis (ANOVA, Tukey) of cytotoxicity over time. Compared are mean values (±SD) of calculated area under the curve. ****, *P = *0.0001.

Because members of the K. oxytoca complex harbor *tnaA* and can produce indole endogenously, differentiating the effects of endogenous versus exogenous indole is necessary to understand environmental regulation of cytotoxin synthesis. Thus, we next investigated the impact of exogenous indole in K. oxytoca AAHC isolate AHC-6 and an isogenic *tnaA* mutant (36), which cannot produce tilivalline without exogenous indole (36). At 18 h of culture, wild-type AHC-6 and the Δ*tnaA* strains produced >50-fold more tilimycin than UCH-1 when grown in media with glucose while showing similar growth (Fig. 4A and B and Fig. 5). Similar to UCH-1, exogenous indole (1 mM) repressed expression of *npsA* and -*B* and tilimycin synthesis in AHC-6 strains (Fig. 4C and D). Tilivalline synthesis remained low in wild-type AHC-6 regardless of whether indole was added, further confirming that the conversion of tilimycin to tilivalline was not a major factor responsible for the reduction in tilimycin. In cultures of the Δ*tnaA* mutant, tilivalline was detected only upon the addition of exogenous indole, supporting the notion that a fraction of tilimycin spontaneously converts to tilivalline in the presence of indole (36).

### Indole mitigates K. oxytoca complex-induced enterotoxicity.

We next evaluated the opposing effects of glucose and indole on UCH-1-induced enterotoxicity. T84 enterocytes were treated with filtered supernatants from UCH-1 grown overnight in LB, LBG, or LBG plus 1.0 mM indole and analyzed by flow cytometry to quantify the percentages of cells undergoing apoptosis. Cells exposed to supernatants from UCH-1 grown in LBG demonstrated a large sub-G1 apoptotic peak indicating fragmented DNA that had leaked from the cells (Fig. 5A). Conversely, supernatants from UCH-1 grown in LB, or LBG plus indole, were much less cytotoxic (Fig. 5A). MS analysis of the corresponding supernatants demonstrated that glucose increased tilimycin synthesis, whereas the absence of glucose resulted in much lower tilimycin levels (Fig. 5A). Although glucose also increased tilivalline synthesis, these levels were substantially lower than those observed for tilimycin (Fig. 5A). On the other hand, the addition of indole to LBG significantly reduced tilimycin synthesis but had minimal effect on tilivalline, which, together with the cytotoxicity results, suggests that tilimycin was the major toxin responsible for inducing cell damage. We next compared the impact of self-produced indole on AHC-6-induced cytotoxicity. Indole-producing AHC-6 and its isogenic Δ*tnaA* mutant with or without a *tnaA*-expressing plasmid in *trans* were cultivated for 50 h *in vitro*. No difference in growth was observed between the strains (Fig. 5B), yet medium conditioned by the indole nonproducer displayed significantly greater cytotoxicity (Fig. 5C and Fig. S2). Higher production was observed earlier during cultivation of the Δ*tnaA* mutant than for the wild-type and complementation strains, and peak activity released in the absence of indole persisted for several hours longer. Given that 100% cell killing was induced in the most cytotoxic fractions (24 h), a further dilution of the media was necessary to resolve interstrain differences (Fig. S2). These data revealed that significantly higher cytotoxic activity was generated in the absence of indole than in its presence.

10.1128/mbio.03752-21.2 FIG S2 Further dilution (1/54) of culture supernatants (from Fig. 5C in the main text) resolves differences in peak cytotoxicity. Shown are reciprocal values (mean ± SD), in percentage, of surviving HeLa cells after treatment with supernatant from K. oxytoca AHC-6, the indole-deficient AHC-6 Δ*tnaA*, and complementing strain AHC-6 Δ*tnaA* (pTnaA) (*n* = 3; measured in triplicates). Statistical comparison (Mann-Whitney) of AHC-6 and AHC-6 Δ*tnaA* was done for each time point. *, *P < *0.05; **, *P < *0.01. Download FIG S2, TIF file, 0.1 MB. Copyright © 2022 Ledala et al. 2022 Ledala et al. https://creativecommons.org/licenses/by/4.0/ This content is distributed under the terms of the Creative Commons Attribution 4.0 International license.

### Indole regulates cytotoxin production *in vivo* and enhances biofilm formation.

To understand further how the induction of cytotoxin synthesis by fermentable carbohydrates could impact microbial communities in the neonatal gut, we assessed the effects of tilimycin on Lactobacillus acidophilus and Bifidobacterium longum, bacterial species that frequently inhabit the gut of breastfed infants (42). In agreement with our previous observations (38), tilimycin, but not tilivalline, demonstrated antibacterial activity (Fig. S3). These results strengthen the notion that production of tilimycin confers a competitive advantage over other bacteria in the gut microflora in the presence of a metabolizable carbon source such as glucose.

10.1128/mbio.03752-21.3 FIG S3 Evaluation of the antimicrobial properties of tilimycin and tilivalline by anaerobic disk diffusion assay. Shown are Lactobacillus acidophilus (ATCC 4356), Cutibacterium acnes (ATCC 6919), and Bifidobacterium longum subsp. *infantis* (UMA272 and UMA299) on various agar media overlaid with DMSO, tilimycin, or tilivalline. D, DMSO (10 μL); T and TV, tilimycin and tilivalline, respectively, with 200 μg of each applied in 10 μL of DMSO. Large zones of inhibition are noted around tilimycin, whereas no effect was observed with tilivalline or DMSO. Download FIG S3, TIF file, 0.5 MB. Copyright © 2022 Ledala et al. 2022 Ledala et al. https://creativecommons.org/licenses/by/4.0/ This content is distributed under the terms of the Creative Commons Attribution 4.0 International license.

**FIG 6:**
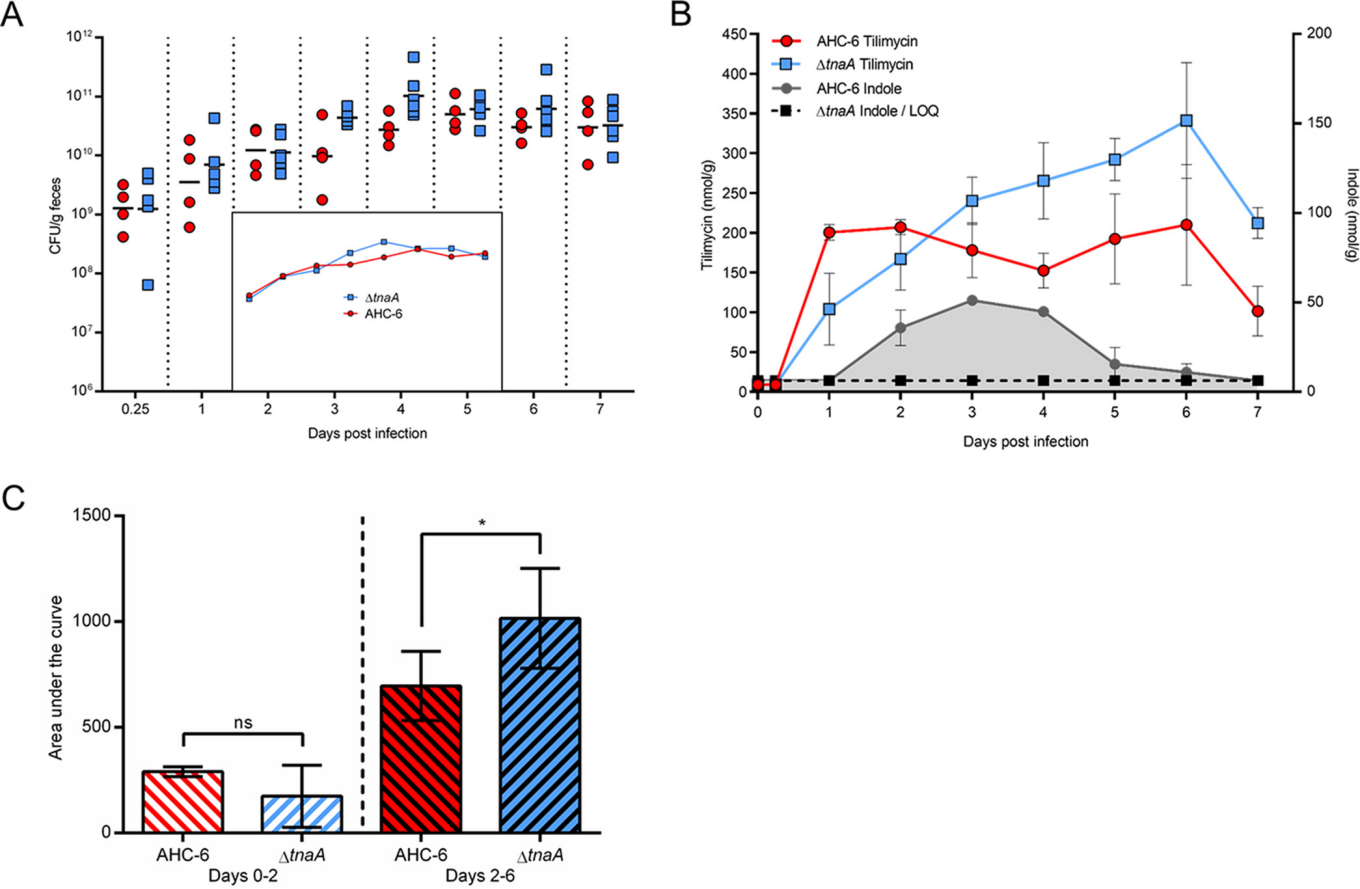
Indole represses tilimycin synthesis *in vivo*. C57BL/6NRj mice were infected with K. oxytoca AHC-6 (*n *= 4) or the indole-deficient Δ*tnaA* strain (*n* = 6). (A) K. oxytoca colonization levels were determined by plating fecal samples on CASO-kanamycin agar. Shown are CFU per gram of feces over time for individual mice colonized with AHC-6 or the Δ*tnaA* strain; black horizontal bars represent the geometric means. The inset shows a simplified comparison of geometric means for both strains over time in the same scale. (B) Fecal tilimycin and indole values were determined by LC-MS daily for each mouse. Plotted are mean ± SEM tilimycin and indole values for each group (AHC-6; Δ*tnaA*) and day. Dashed black lines represent the limit of quantification for indole (LOQ) and simultaneously indicate lack of quantifiable indole values for the Δ*tnaA* group. (C) Statistical significance of differences in total tilimycin amounts (area under the curve) generated by AHC-6 and the Δ*tnaA* mutant before (days 0 to 2) and after (days 2 to 6) indole production was determined by Mann-Whitney U test. *, *P = *0.0333.

We next employed an animal model (35) to evaluate the capacity of indole to regulate enterotoxin production *in vivo*. To recapitulate antibiotic-induced dysbiosis in patients, the murine microbiota was depleted by administration of water containing amoxicillin-clavulanic acid (Curam). Mice then were gavaged with equivalent numbers of AHC-6 or the Δ*tnaA* mutant and colonized over the course of infection (Fig. 6A). In this colonization model without a nonsteroidal anti-inflammatory drug (NSAID), mice are infected but do not develop colitis, which allows mice to survive for reliable measurements of fecal indole and tilimycin concentrations (35). The fecal metabolites of colonized mice were measured daily for 1 week postinfection using MS as described previously (43). Indole remained under the limit of quantitation throughout the experiment for animals colonized with the Δ*tnaA* mutant (Fig. 6B). Tilimycin was detected in samples from this cohort 24 h postinfection, and levels increased continuously and evenly until reaching a maximum on day 6. In contrast, a moderately high peak of tilimycin was present in stool of animals colonized with AHC-6 already on day 1, when no quantifiable indole was measured. After the emergence of indole on day 2, no further increase in tilimycin was observed until depletion of indole levels became apparent after day 4 (Fig. 6B). The impact of the observed regulation on tilimycin accumulation over time revealed significantly less tilimycin in wild-type colonized mice than with the mutant when indole was available (days 2 to 6) (Fig. 6C). These data support the conclusion that the presence of luminal indole reduced the production of tilimycin *in vivo*. Conceivably, AHC-6 enters a quiescent state late in infection. In support of this possibility, the *tnaA* mutant demonstrated increased biofilm formation when grown in the presence of increasing concentrations of indole (Fig. S4).

10.1128/mbio.03752-21.4 FIG S4 Exogenous indole induces biofilm formation by AHC-6 Δ*tnaA*. Shown are the absorbance values (OD_570_) after 48 h of incubation at 37°C, presented as mean values ± standard errors of the means (*n *= 3). Analysis was done by Kruskal-Wallis followed by Dunn’s posttest. *, *P ≤ *0.05; **, *P ≤ *0.01 compared to value with vehicle. Download FIG S4, TIF file, 0.05 MB. Copyright © 2022 Ledala et al. 2022 Ledala et al. https://creativecommons.org/licenses/by/4.0/ This content is distributed under the terms of the Creative Commons Attribution 4.0 International license.

### Tilivalline, but not tilimycin, is a PXR ligand and agonist.

**FIG 7:**
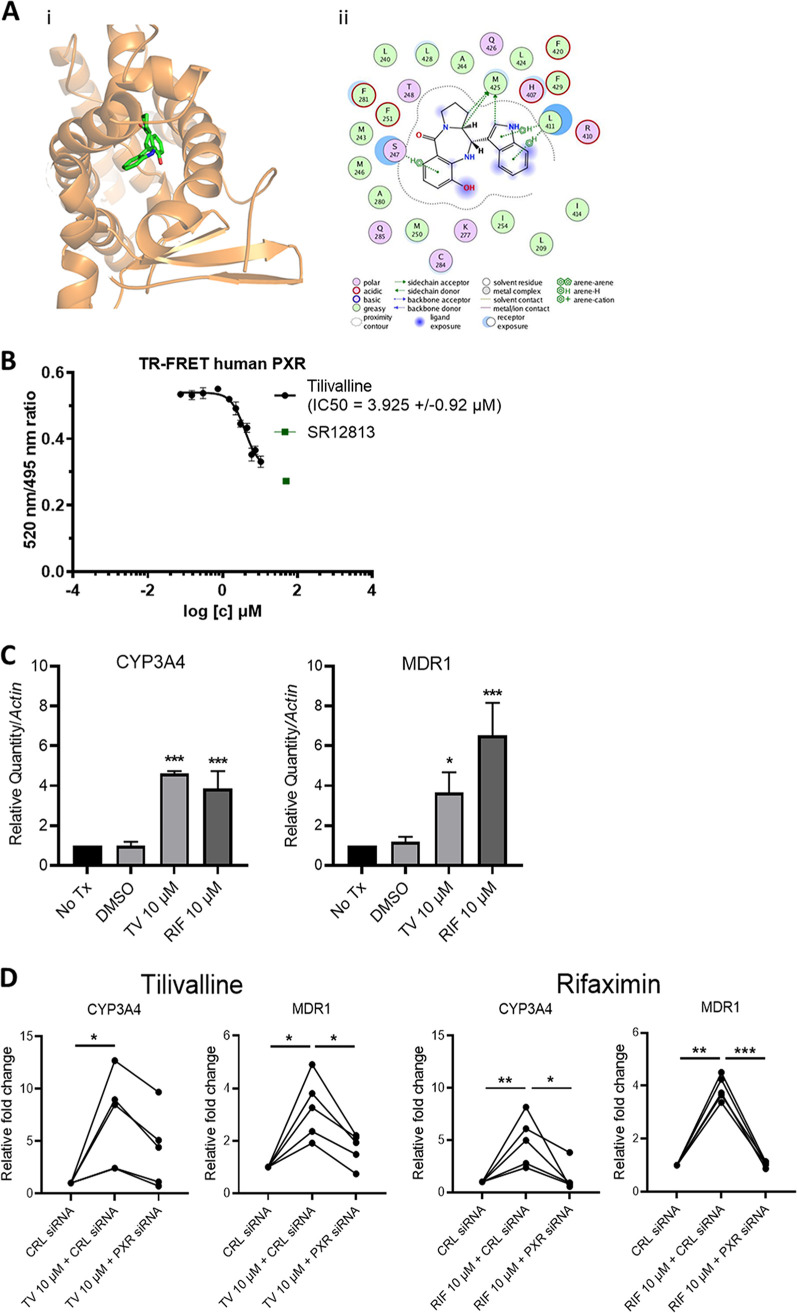
Tilivalline is a PXR agonist. (A) Docked complex of tilivalline with PXR. (i) Three-dimensional representation with PXR represented as ribbons and colored orange, while tilivalline is represented as licorice sticks and colored atom type (C, green; N, blue; and O, red). Hydrogen atoms have been removed for clarity. (ii) Schematic representation of the interactions of tilivalline with the ligand binding domain of PXR drawn using the ligand interaction module of MOE. The schematic legend at the bottom explains the types of interactions. (B) LanthaScreen TR-FRET PXR competitive binding assay. TR-FRET ratios (520/495 nm) are plotted against concentrations of tilivalline and SR12813. Half-maximal inhibitory concentrations (IC_50_s) were obtained from interpolated standard curves (sigmoidal, 4PL, variable slope); error bars show standard deviations (*n *= 3 or 4). (C) Relative mRNA levels for *cyp3A4* and *mdr1* in LS180 cells treated with tilivalline (TV) (10 μM), rifaximin (RIF) (10 μM; positive control), or DMSO (0.1% [vol/vol]; vehicle) determined by RT-qPCR. Data represent mean values ± standard errors of the means (*n *= 3 or 4). Tx, treatment. (D) Changes in *cyp3A4* and *mdr1* mRNA levels following treatment with TV (10 μM) or RIF (10 μM) in LS180 cells transfected with control (CRL) or PXR siRNA (*n *= 5). For panels C and D, statistical analysis was done by one-way ANOVA with multiple comparisons. *, *P ≤ *0.05; **, *P ≤ *0.01; ***, *P ≤ *0.001.

PXR, a host-derived master transcription factor and xenobiotic sensor involved in detoxification of small molecules, is well expressed in the intestinal epithelium, where it binds to indole analogs/metabolites (11). At a high dose, tilivalline, an indole analog, is reported to induce mitotic arrest via its microtubule-stabilizing activities (38). To investigate if PXR could contribute to the modulation of tilivalline-induced toxicity, we first evaluated whether tilivalline could be a PXR ligand (44). Molecular docking predicted that tilivalline docks to the agonist ligand binding domain (LBD) of human PXR (hPXR) (docking score of 50.86) (Fig. 7Ai). A comparison of the binding modes of indole and tilivalline at the ligand binding domain shows that although they bind to the same promiscuous binding pocket, with some overlapping residues, the interactions that directly contribute to the binding affinity for the ligands are predicted to be different. Interactions predicted to contribute to the high docking score of tilivalline in the ligand binding domain include aryl-hydrogen interactions with Ser247 and Leu411, electrostatic interactions with Met425, His407, Arg410, Lys277, and Cys284, and hydrophobic interactions with Phe251, -281, and -429, Leu240 and -428, Ala280 and -244, Ile 254 and -414, and Met243, -246, and -250 (Fig. 7Aii). The docking prediction then was confirmed by time-resolved fluorescence resonance energy transfer (TR-FRET) PXR competitive binding assay with tilivalline concentrations ranging from 0.1 to 3.5 μg/mL (0.30 to 10.50 μM). In three independent experiments, we observed clear dose-dependent competition curves indicating that tilivalline is a PXR ligand (Fig. 7B). Conversely, tilimycin, which lacks an indole moiety (36), demonstrated no interaction with PXR (Fig. S5A).

10.1128/mbio.03752-21.5 FIG S5 Tilimycin is not a PXR ligand, whereas tilivalline is specific for PXR in intestinal cells but not hepatocytes. (A) Tilimycin demonstrated no binding in the LanthaScreen TR-FRET PXR competitive binding assay. TR-FRET ratios (520/495 nm) are plotted against concentration of tilimycin and SR12813. IC_50_s were obtained from interpolated standard curves of two independent experiments. Error bars indicate standard deviations (*n *= 2). (B) Tilivalline induced expression of PXR target genes in cultured human enterocytes in a dose-dependent manner. Relative mRNA levels for *cyp3A4* and *mdr1* in LS180 cells treated for 48 h with increasing concentrations of tilivalline (TV) or rifaximin (RIF) (10 μM, positive control) were determined by RT-qPCR. Here, values were compared to the case with no treatment (Tx) (*n *= 3). (C) Tilivalline induced expression of PXR-target genes in murine intestinal tissues. Fold change of mRNA levels for *cyp3a11*, *Abcb1a*, and *Nr1i2* were determined by RT-qPCR, for intestinal organoids treated *ex vivo* with tilivalline (20 μM) or tilimycin (TM) (0.5 to 0.75 μM) (*n *= 2 donors). (D) Minimal agonist effect of tilivalline for PXR target genes in primary human hepatocytes. Cultured cells from 3 different donors were treated for 24 h with tilivalline (3 to 30 μM), rifampicin (RIF) (10 μM; positive control) or combinations as indicated. Relative mRNA levels for *cyp3A4* and *mdr1* were determined by RT-qPCR (*n *= 3). Data represent mean values ± standard errors of the means. Statistical analysis was done by one-way ANOVA with multiple comparisons. *, *P ≤ *0.05; **, *P* ≤ 0.01; ***, *P ≤ *0.001. Download FIG S5, TIF file, 0.5 MB. Copyright © 2022 Ledala et al. 2022 Ledala et al. https://creativecommons.org/licenses/by/4.0/ This content is distributed under the terms of the Creative Commons Attribution 4.0 International license.

We next evaluated the capacity of tilivalline to activate PXR via the induction of PXR-responsive genes, CYP3A4 and MDR1, in LS180 enterocytes (45). Addition of tilivalline (10 μM) to the cells induced the PXR-responsive genes at levels similar to those of the model PXR ligand rifaximin (Fig. 7C), indicating that tilivalline is a PXR agonist. Tilivalline further upregulated CYP3A4 and MDR1 in a dose-dependent manner (Fig. S5B). Similar upregulation of PXR-responsive genes was observed in the crypts of murine intestinal organoids following cultivation in the presence of 20 μM tilivalline (Fig. S5C). To confirm that the induction of CYP3A4 and MDR1 was PXR specific and did not involve other nuclear receptors (46), LS180 cells were treated with PXR small interfering RNA (siRNA) or control siRNA and then exposed to tilivalline. PXR siRNA resulted in an 80% reduction in endogenous PXR expression. As with rifaximin, a known PXR agonist (47), under conditions of reduced PXR expression, tilivalline induced significantly less upregulation of PXR-responsive genes (Fig. 7D). To evaluate the tissue specificity of tilivalline-PXR interactions, we evaluated the capacity of tilivalline to upregulate PXR-responsive genes in primary human hepatocytes. In these cells, tilivalline resulted in negligible induction of CPY3A4 and MDR1 (Fig. S5D), indicating that tilivalline is an intestinal, but not liver, PXR agonist, as reported for other indole scaffold PXR ligands (48).

### PXR modulates tilivalline-induced enterocyte toxicity.

**FIG 8:**
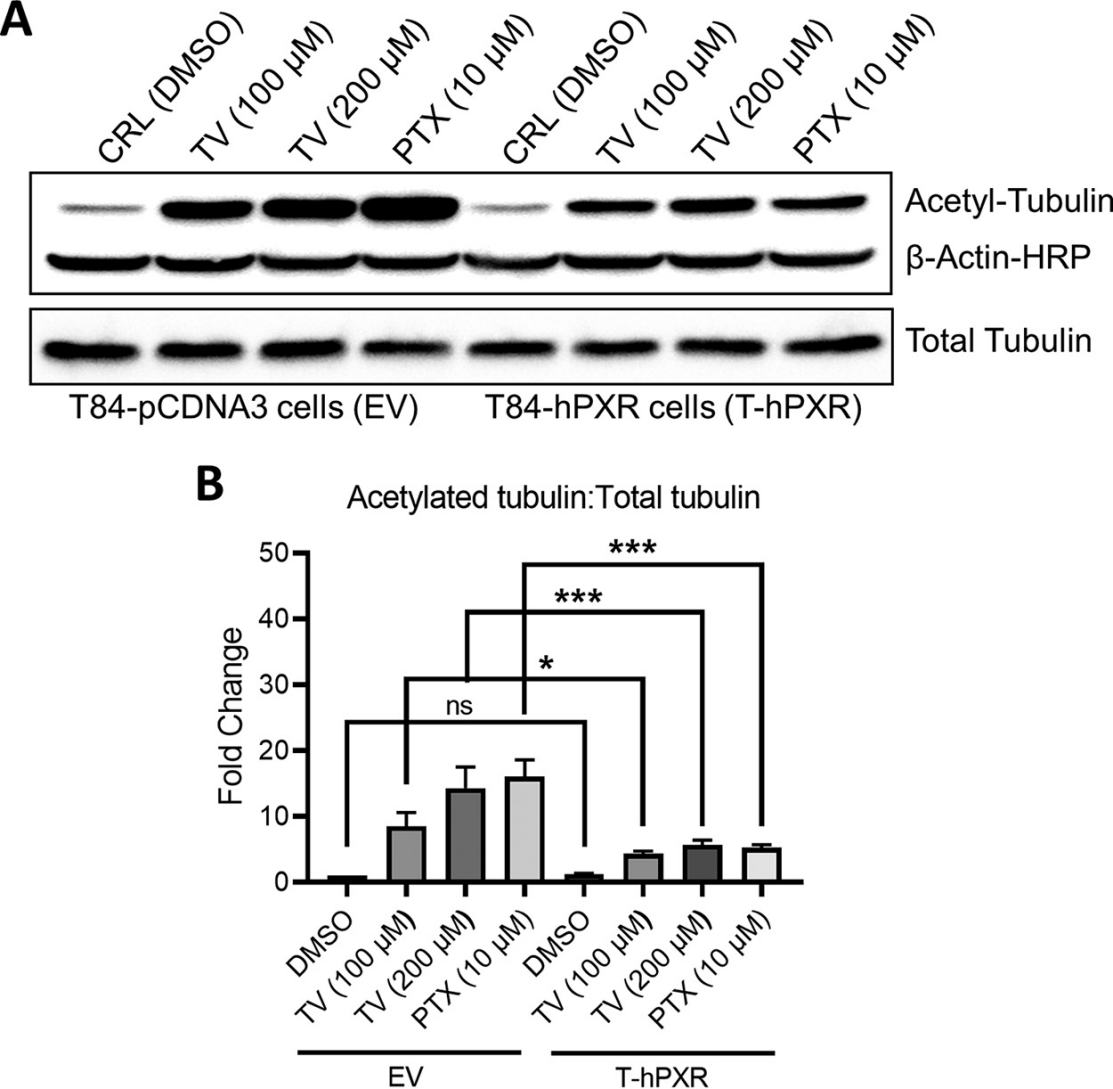
PXR abrogates tilivalline-induced enterocyte tubulin acetylation. (A) Immunoblot of acetylated tubulin, total tubulin, and β-actin in T84 cells stably transfected with empty vector pCDNA3 (EV) or human PXR (hPXR). Cells were treated with tilivalline (100 μM and 200 μM) or paclitaxel (PTX) (10 μM), an established tubulin-acetylating/polymerizing agent whose toxic effects are modulated by PXR; cell lysates were transferred to nitrocellulose membranes and probed with antibodies specific for anti-acetylated tubulin, total tubulin, and β-actin. (B) Values of band intensities in EV and T-hPXR T84 cells with or without various treatments. The data represent the mean values ± standard errors of the means (*n *= 3). Statistical analysis was done by one-way ANOVA with multiple comparisons. *, *P ≤ *0.05; ***, *P ≤ *0.001.

Tilivalline induces tubulin acetylation/polymerization and disrupts enterocyte renewal, which is in contrast to the DNA-damaging effects of tilimycin (38). As a result, tubulin-based assays represent more sensitive and physiologic assessments of tilivalline-induced cellular injury than measurements of apoptosis (Fig. S6). To determine if PXR could modulate tilivalline effects, T84 enterocytes were stably transfected with PXR or an empty vector. Baseline expression of PXR in wild-type T84 cells is very low (49); following transfection, PXR expression was increased 4- to 5-fold. Tilivalline (100 to 200 μM) then was applied to the cells; as a positive control, the drug paclitaxel (Taxol; 10 μM), an established tubulin acetylating/polymerizing agent whose toxic effects also are modulated by PXR (50, 51), was applied to the cells in separate wells. Following 16 h of exposure, the T84 cells were harvested and immunoblotting was performed to determine the fraction of acetylated α-tubulin versus total α-tubulin as an indicator of microtubule stability (52). In the empty-vector cells, tilivalline induced tubulin acetylation in a manner similar to that of paclitaxel, whereas the fraction of acetylated tubulin was significantly reduced in PXR-transfected cells, indicating that PXR mitigates the effects of this enterotoxin (Fig. 8).

10.1128/mbio.03752-21.6 FIG S6 Tilivalline induces low levels of caspase-3 activity which are not significantly reduced by PXR. Caspase-3 activity was measured in T84 enterocytes stably transfected with empty vector pCDNA3 (EV) or human PXR (hPXR), following 48 h of exposure to tilivalline (50 μM), DMSO, or no treatment (*n *= 3). Data represent mean values ± standard errors of the means. Statistical analysis was done by Mann-Whitney U test. Download FIG S6, TIF file, 0.2 MB. Copyright © 2022 Ledala et al. 2022 Ledala et al. https://creativecommons.org/licenses/by/4.0/ This content is distributed under the terms of the Creative Commons Attribution 4.0 International license.

## DISCUSSION

Commensal microbes and host factors play a crucial role in regulating the human intestinal ecosystem and maintaining homeostasis. Microbe-microbe and host-microbe interactions are further influenced by environmental changes, such as nutrient availability, which can shape the behaviors and signaling molecules produced by the resident microbiota (53). The context in which a microbe exhibits different characteristics also can have a dramatic impact on determining if it is friend or foe (54). Here, we report a novel communication network involving the regulation of *K. grimontii* and K. oxytoca, two closely related species regarded as a human gut commensals but also with the capacity to induce toxin-mediated colitis (20, 21, 28). Availability of metabolizable carbohydrates, environmental indole concentrations, and host PXR were found to modulate cytotoxic effects in an integrated manner. This study expands the growing body of literature showing that indole is important signaling molecule that reduces pathogenicity (5–9, 55) while also incorporating unique elements of microbial, metabolic, and host cross talk in the modulation of virulence.

K. oxytoca is commonly isolated from humans and belongs to the early colonizers of the infant gut microbiota (25). Cytotoxin-producing strains are the causative agent of AAHC (20, 21) and have been identified in premature infants with and without NEC (22, 34). Several other studies have implicated Klebsiella spp. in NEC but failed to discriminate among species (56, 57) or did not differentiate between toxin-positive and toxin-negative strains (58). Recent extended genomic analyses of Klebsiella spp. have subdivided members of the K. oxytoca complex into various phylogroups, with strains of K. michiganensis, K. grimontii, and K. pasteurii, along with K. oxytoca, reported to contain the biosynthetic gene cluster (BGC) encoding the enzymatic pathway for tilimycin synthesis (34). Given this recent reclassification, we performed WGS and ANI/OrthoANI comparisons on UCH-1, isolated from a patient with NEC, and found that it matched closest with *K. grimontii*. Similar to K. oxytoca, isolates of *K. grimontii* have been recovered from the fecal stream of subjects with AAHC and asymptomatic carriers (28). *K. grimontii* harboring the tilimycin BGC also has been recovered from preterm (33, 34) and term (26) infants.

The link between carbohydrate metabolism and virulence determinants has been demonstrated for several other enteric pathogens, including cytotoxin-producing *Clostridium* species (59). Here, we demonstrate that the presence of a fermentable carbohydrate (i.e., glucose) increases toxin synthesis and cytotoxicity induced by species of the K. oxytoca complex. These results agree with a prior study which demonstrated variations in cytotoxicity using culture supernatants from K. oxytoca complex isolate MH43-1 from different culture conditions (37). In some patients taking β-lactam antibiotics, overgrowth of one or more members of the K. oxytoca complex can result in AAHC (20, 21). While expansion of this microbe is generally regarded as the primary driver of AAHC, it remains to be determined if the release of fermentable carbohydrates by commensal bacteria upon antibiotic treatment is contributory, as reported for C. difficile colitis (59). A role for K. oxytoca complex-related infections also has been reported for individuals with diabetes (60); while it is not clear if the isolates were toxin positive, the presence of this toxin-producing gene cluster could complicate the management of infections caused by multidrug-resistant strains. Presumably, toxin-positive species increase cytotoxin synthesis in response to fermentable carbohydrates to enhance their ability to compete for nutritional resources. As demonstrated previously (38), and confirmed in the present study, tilimycin exhibits antibacterial activity against inhabitants of the gut microbiota. It is possible, therefore, that intestinal injury invoked by K. oxytoca complex cytotoxins represent collateral damage.

Malabsorption of carbohydrates has been associated with the development of NEC (61) and enhances intestinal damage in animal models of NEC (62); however, these linkages have not been attributed to the pathogenic potential of a specific commensal microbe (4). Many preterm formulas contain glucose in the form of corn syrup solids. The preterm gut has immature glycosidase activity, which increases the risk of carbohydrate malabsorption (63). Thus, the nondigested carbohydrates in the preterm gut could serve as a preferred carbon source for tryptophanase producers, thereby decreasing luminal indole levels and inciting cytotoxicity by members of the K. oxytoca complex. Interestingly, fecal tryptophan levels were reported to be decreased in preterm infants exposed to early antibiotics (64), and several studies suggest that antibiotic use in preterm infants increases the risk of NEC (65, 66). Members of the K. oxytoca complex also utilize a broader array of sugars than other Klebsiella spp., which may provide the fermentative energy to support toxin production and promote colonization resistance of other enteric bacteria (67, 68). Perturbations in the gut microbiome leading to excessive Toll-like receptor 4 (TLR4) stimulation have been suggested as an inciting event in NEC pathogenesis (69, 70), whereas others have challenged the notion that NEC represents a single disease entity (71). Our findings suggest that cytotoxin production by members of the K. oxytoca complex represents an additional pathway leading to mucosal disruption in the preterm gut.

Indole and indole metabolites are increasingly recognized as interspecies and interkingdom signaling molecules that reduce pathogenicity (55). The results obtained from *npsA* and -*B* transcriptional experiments, MS cytotoxin measurements, and cytotoxicity assays extend these observations to include the regulation of virulence by the K. oxytoca complex. Furthermore, the data obtained using the murine model confirm that these signaling pathways respond similarly *in vivo*. In the gut lumen, microbes sense and respond to indole (5, 6, 72), while host cells absorb indole to strengthen barrier integrity and facilitate beneficial host-microbe interactions (10). Among these interactions, the induction of enterocyte PXR facilitates anti-inflammatory and detoxification responses (12, 13). Indole is generated as a by-product of the conversion of tryptophan to pyruvate by tryptophanase (TnaA), an enzyme that is ubiquitous among gut bacteria (18). As demonstrated in the present study, the availability of a preferred carbon source, such as glucose, eliminates the need for bacteria to use this tryptophan conversion pathway; consequently, expression of *tnaA* is repressed and indole production decreases. Alternatively, in the absence of fermentable carbohydrate, *tnaA* is expressed and indole production is high. The general utility of indole as a signaling molecule for bacteria under different environmental conditions is well described (73). It is possible that K. oxytoca complex members utilize luminal indole as a barometer for nutrient availability. The production of secondary metabolites such as tilimycin is energetically costly. Repression of *npsA* and -*B* by indole may minimize energy expenditure and promote mechanisms of persistence, such as biofilm formation (74, 75), while living in a dense ecosystem with limited nutrients; conversely, when there is greater access to nutrients, such as during antibiotic-induced dysbiosis or carbohydrate malabsorption, derepression of *npsA* and -*B* by low luminal indole levels may confer a competitive edge.

In the analysis of culture supernatants from *K. grimontii* (UCH-1) and K. oxytoca (AHC-6), grown in LB with added glucose, both tilimycin and tilivalline were detected; however, concentrations of tilimycin far exceeded those of tilivalline, similar to what is reported for fecal contents of humans with AAHC and the murine model of AAHC using AHC-6 (38). Furthermore, results from the apoptosis experiments performed with UCH-1 grown with and without exogenous indole strongly suggest that tilimycin is the major cytotoxic culprit. Thus, an additional facet of indole regulating K. oxytoca complex pathogenicity is the conversion of the potent toxin tilimycin to the less toxic product tilivalline.

Indoles and indole analogs/metabolites have been shown to interact with mammalian nuclear receptors that modulate host physiologic and inflammatory responses (11). To assess the host’s role in regulating responsiveness to tilimycin and tilivalline, we investigated their binding and agonist activity to PXR, a ligand-activated transcription factor and xenobiotic sensor well known for its role in detoxification, drug metabolism, and regulation of intestinal inflammation (12, 13). In this capacity, PXR functions as a key modulator of inflammatory bowel disease (76) and experimental NEC (77). We found that tilivalline, but not tilimycin, which lacks the indole moiety, functioned as an intestine-specific PXR agonist and upregulated PXR-dependent detoxification genes. This interaction also limited the toxic effects of tilivalline on human enterocytes by mitigating tubulin acetylation. Hence, PXR works in a coordinated manner with commensal indole producers following the conversion of tilimycin to tilivalline. Given the wide range of effects that PXR exerts on intestinal homeostasis and host metabolism, these results support the notion of a significant interplay between microbial metabolites and gut physiology (78). Collectively, these results also indicate that nutrient availability, which profoundly effects the metabolic status of tryptophanase producers, is an important element of this network.

The comparative analysis of *K. grimontii* strain UCH-1 and K. oxytoca strain AHC-6 revealed that expression of *npsA* and -*B* and tilimycin concentrations were dramatically higher in AHC-6. This strain was originally cultured from an adult subject with AAHC (20), whereas UCH-1 was from a preterm infant with NEC (22). Additional studies are needed to determine if differential cytotoxin production is a consistent feature among phylogroups and how the context of cytotoxin synthesis, such as within the preterm gut versus mature gut, impacts pathogenicity. Polymorphisms in upstream regulatory regions could explain the transcriptional differences, which, in turn, impacts cytotoxin biosynthesis, as with C. difficile “supertoxin” producers (79). Regardless, both isolates were found to respond to carbohydrate and indole in an analogous fashion, extending these observations across the two phylogroups.

In summary, we have demonstrated a novel indole-based signaling pathway involving intestinal, bacterial, and nutritional components in the regulation of K. oxytoca complex pathogenicity. Deciphering the dysbiotic environmental conditions, genetic diversity of specific strains, and contextual factors are important parameters for understanding how commensal microbes alter their behaviors and cause disease. Such studies are needed to unravel how these factors contribute to K. oxytoca complex-induced intestinal pathology in preterm infants with NEC and older children and adults who develop AAHC.

## MATERIALS AND METHODS

### Bacterial culture conditions.

For routine culture, all strains were grown in Luria-Bertani broth or agar (LB; BD Biosciences, Franklin Lakes, NJ) and all media were filter sterilized. When required, media were supplemented with glucose (2.5 g/L), indole (Sigma, St. Louis, MO) in 0.1% (vol/vol) *N*,*N*-dimethylformamide (DMF), or DMF alone (vehicle). Bacterial cultures were grown overnight in 5 mL of LB broth, harvested by centrifugation at 5,000 rpm, washed once in 5 mL of sterile saline, and suspended in fresh LB broth. This preparation was used to inoculate prewarmed medium to 0.06 unit (absorbance at 600 nm) to prepare the primary experimental culture(s). Growth (optical density at 600 nm [OD_600_]) was measured every hour for 12 h while cultured in 10:1 (vol/vol) flask-to-medium ratios at 37°C with shaking at 225 rpm.

### Bacterial RNA purification and analysis.

Quantities of 5 U (OD_600_) of culture samples were mixed with twice the volume of bacterial RNAprotect (Qiagen, Valencia, CA) and centrifuged for 10 min at 5,000 rpm. The supernatants from these samples were discarded and cell pellets were mixed with 1 mL of TRIzol (Invitrogen, Carlsbad, CA), transferred to lysing matrix B tubes (MP Biomedicals, Irvine, CA), and subjected to lysis using Disruptor Genie (Scientific Industries, Bohemia, NY) for 4 min at 3,000 rpm. The cell lysate was processed using an RNeasy kit (Qiagen) per the manufacturer’s instructions. The concentration and purity of isolated RNA were determined using a NanoDrop 2000 UV-visible (UV-Vis) spectrophotometer (Thermo Fisher Scientific, Waltham, MA).

### cDNA synthesis and RT-qPCR.

A total of 500 ng of RNA was used to prepare cDNA using the iScript master mix (Bio-Rad, Hercules, CA) reverse transcriptase reaction protocol (5 min at 25°C, 20 min at 46°C, and 1 min at 95°C) in a 20-μL volume. Following this reaction, each sample was diluted 10-fold and 5 μL was used for real-time quantitative PCR (RT-qPCR) in a total reaction volume of 25 μL containing 12.5 μL of 2× SsoAdvanced Sybr green supermix (Bio-Rad) and 8.75 pmol of each primer. Primers for the target genes and the internal reference gene (*recA*) are listed in Table S2. The reaction and preliminary data analysis were carried out on a CFX96 real-time PCR detection system and Bio-Rad CFX Manager software version 3.0 (Bio-Rad). Transcripts were plotted as target copies per 100 copies of the reference gene (80). For human intestinal cell lines and murine intestinal organoids, transcript copy numbers were normalized to the β-actin gene or *Ppib*, *Tbp*, and *Gusb*, respectively.

10.1128/mbio.03752-21.8 TABLE S2 Oligonucleotide primers used in this study. Download Table S2, XLSX file, 0.01 MB. Copyright © 2022 Ledala et al. 2022 Ledala et al. https://creativecommons.org/licenses/by/4.0/ This content is distributed under the terms of the Creative Commons Attribution 4.0 International license.

### Detection of tilimycin and tilivalline in bacterial culture supernatants by UPLC-MS/MS.

Samples were analyzed at the University of Connecticut Center for Environmental Sciences and Engineering (Storrs, CT) using a Waters Acquity ultraperformance liquid chromatograph (UPLC) coupled with an Acquity TQD tandem mass spectrometer (Waters Co., Milford, MA) as described previously (22). A total of 450 μL of test or quality control samples were spiked with 25 μL internal standard solution and 25 μL of methanol. An Acquity UPLC BEH C18 (1.7 μm, 2.1 × 50 mm) column, maintained at 25°C and with a sample injection volume of 10 μL on a 20-μL loop, was utilized for analyte separation. Analyte signal optimization was performed using the Waters IntelliStart, whereas analyte detection, quantification, and statistical analysis were performed using the Waters QuanLynx within MassLynx software v.4.2 in electrospray ionization (ESI) plus tandem MS (MS/MS) mode.

### Flow cytometry.

T84 enterocytes were plated at a density of 2 × 10^5^ to 3 × 10^5^ per well at 37°C with 5% CO_2_. Filtered bacterial supernatants were added at a 1:1 dilution for 72 h. Following incubation, the cells were fixed, permeabilized, and stained with propidium iodide as described previously (22). Flow cytometry was performed using an LSRII (BD Biosciences), and the data were analyzed using FlowJo software (Tree Star Inc., Ashland, OR).

### Cytotoxicity of AHC-6 culture supernatants.

HeLa cells (0.8 × 10^5^ to 1.0 × 10^5^) were seeded in 96-well plates containing Dulbecco’s modified Eagle’s medium (DMEM) supplemented with 10% fetal bovine serum (FBS), 100 μg/mL of penicillin, and 100 μg/mL of streptomycin and allowed to attach for 24 h. K. oxytoca strains were cultured in tryptic soy (CASO) broth with shaking for 50 h, and samples were taken during cultivation. Conditioned media were cleared by centrifugation (5 min, 10,000 rpm) and filtered through a sterile 0.2-μm syringe filter, and 50 μL of 1:27 dilutions was added per well. After 2 days of incubation at 37°C with 5% CO_2_, cell survival was determined via MTT [3-(4,5-dimethylthiazol-2-yl)-2,5-diphenyltetrazolium bromide] assay as described previously (24).

### Murine infection model.

Animal experiments were performed as previously described (35). Adult female C57BL/6NRj mice with specific-opportunistic-pathogen-free status were purchased from Janvier labs and housed under specific-pathogen-free conditions in individually ventilated cages. Mice (age 8 weeks) were administered amoxicillin-clavulanic acid in drinking water to final concentrations of 0.4 mg/mL of amoxicillin and 0.04 mg/mL of clavulanate 24 h before infection with 1 × 10^7^ CFU of K. oxytoca AHC-6 *aphA* (Kan^r^) (35) or the Δ*tnaA* mutant (36). Fecal pellets were collected 6 h after gavage and daily thereafter. Quantification of bacteria in inocula and in feces of colonized animals (CFU per gram of stool) was performed by plating of serial dilutions on tryptic soy (CASO) agar (40 μg/mL of kanamycin). Fecal metabolites were extracted and quantified as previously described (43).

### Test substances and antibacterial assay.

Tilimycin and tilivalline were synthesized by the Chemical Synthesis and Biology Core Facility, Albert Einstein College of Medicine (Bronx, NY), and Graz University of Technology. Tilivalline also was purchased (Santa Cruz, Dallas, TX). The antibacterial effects of tilimycin and tilivalline were determined using a disk diffusion assay as previously described (81) at the concentrations indicated in the figure legends.

### Microtiter plate biofilm formation assay.

Biofilm formation by AHC-6 *ΔtnaA* strain was assessed by microtiter plate assay (82). Briefly, 20 μL of bacterial suspension at an OD_600_ of 0.5 was inoculated in each well containing 180 μL of LB broth with increasing concentrations of indole. Following incubation for 48 h, the wells were decanted and washed, and 0.1% crystal violet solution was added for 15 min. The wells then were washed and dried at room temperature for up to 24 h. Finally, the cell-bound crystal violet was dissolved in 30% acetic acid in water. Biofilm growth was quantified using a microplate reader (Bio-Rad) at 570 nm using 30% acetic acid in water as the blank.

### Molecular docking analysis of tilivalline.

The PXR crystal structure (PDB code 1M13 [83]) was prepared for docking studies of tilivalline as described previously (84). The structure was then energy minimized and refined using molecular dynamics simulations using Amber charges and Amber force field as adopted in the modeling program MOE (Molecular Operating Environment; version 2016.0801). Tilivalline was modeled using the ligand builder module of MOE and optimized for geometry. The ligand binding domain of PXR has been previously validated to bind indole-like molecules (44). Hence, tilivalline was docked to the ligand binding domain of PXR using GOLD suite version 5.5.0 (CCDC, Cambridge, UK) (85); 20 independent runs were performed to completely sample the receptor and ligand conformational space.

### hPXR competitive ligand binding assay.

hPXR competitive ligand binding assay was carried out using a LanthaScreen time-resolved fluorescence resonance energy transfer (TR-FRET) PXR competitive binding assay kit (Invitrogen, Carlsbad, CA) according to the manufacturer’s instructions. Test compounds (tilivalline and tilimycin), SR12813 (1 μM; positive control), or dimethyl sulfoxide (DMSO; 1%) to serve as a vehicle control was incubated with the Fluormone PXR (SXR) green (fluorescein-labeled PXR ligand) (40 nM), human PXR-LBD (glutathione *S*-transferase [GST] labeled) (5 nM), LanthaScreen Tb-anti-GST antibody (10 nM), and dithiothreitol (50 nM) mixed in TR-FRET PXR (SXR) assay buffer. The assay was carried out in a 384- or 96-well plate, and results were quantified using a BMG LABTECH PHERAstar fluorescent plate reader or Tecan Infinite F200 Pro plate reader (Schoeller Instruments, Czech Republic) using an excitation wavelength of 340 nm, emission wavelengths of 490 nm for terbium and 520 nm for fluorescein, a delay time of 100 μs, and an integration time of 200 μs. Half-maximal inhibitory concentration (IC_50_) for a test compound was determined from the calculated TR-FRET ratio for emission (520:495) and dose-response curve.

### Assays with PXR agonists.

PXR-expressing LS180 enterocytes (45) were plated at a density of 5 × 10^5^/well in 12-well plates. Twenty-four hours later, cells were treated with DMSO (0.1% [vol/vol]; vehicle), tilivalline, and rifaximin at the concentrations indicated in the figure legends. Drug-containing medium was renewed at 24 h. RNA was isolated at 48 h and processed for gene expression as described above. Murine small intestinal crypts were isolated from C57BL/6J mice as previously described (86). Basolateral treatment of intestinal organoids was performed by adding compounds with final solvent concentrations of 0.3%. Plates were incubated at 37°C with 5% CO_2_, and media and treatment were renewed every 2 to 4 days. RNA was isolated from 100 to 1,000 organoids/treatment on day 7 as described above. Primary human hepatocytes in monolayer batches Hep22001014 (male, 76 years, unknown ethnicity) and Hep22001015 (male, 72 years, unknown ethnicity) were purchased from Biopredic International (Rennes, France). Primary human hepatocyte culture from multiorgan donor LH79 (male, 60 years, Caucasian) was prepared at the Faculty of Medicine, Palacky University Olomouc. Primary human hepatocyte cultures were cultured in serum-free ISOM medium.

### siRNA transfection.

hPXR knockdown in LS180 cells was performed by reverse transfection of small interfering RNA (siRNA) according to the manufacturer’s (Dharmacon, Lafayette, CO) instructions. Briefly, 50 nM ON-TARGET SMARTpool PXR siRNA (L-003415-00-005) or nontargeting siRNA (D-001810-01-05) duplex was complexed with 2.5 μL of Lipofectamine RNAiMAX (Invitrogen, Waltham, MA) in Opti-MEM reduced-serum medium and added to the 12-well plate. A total of 3 × 10^5^ cells then were mixed with siRNA complex in wells and incubated at 37°C with 5% CO_2_. The PXR knockdown efficiency, determined by qPCR, was greatest at 48 h (80 to 85% of control). At 48 h posttransfection, the cells were treated with DMSO (0.1%), 10 μM tilivalline, or 10 μM rifaximin (Sigma) for another 48 h. At the end of drug treatment, mRNA was extracted and target gene expression was determined using RT-qPCR as described above.

### Cell culture and generation of stable clones.

LS180 cells were maintained in modified Eagle’s minimal essential medium (EMEM; Invitrogen) supplemented with 10% heat-inactivated FBS, 2 mM l-glutamine, 1% nonessential amino acids (NEAA), 1 mM sodium pyruvate, and 25 mM HEPES without antibiotics at 37°C with 5% CO_2_. T84 cells were maintained in DMEM–F-12 (1:1) supplemented with 5% FBS, 2 mM l-glutamine, and no antibiotics at 37°C with 5% CO_2_. For transfection, T84 cells were plated in 6-well plates at 7 × 10^5^ per well. After 24 h, the cells were transfected with pcDNA3-hPXR or vector plasmid using Lipofectamine-LTXplus (Thermo Fisher Scientific). After 72 h of transfection, the cells were selected with 0.8 mg/mL of G418 for 21 days to establish stable clones. The expression level of PXR was examined with a TaqMan gene expression assay kit (PXR, Hs011265_g1, and ACTB, Hs01060665_g1). Stable clones were maintained with a medium containing 0.4 mg/mL of G418.

### Immunoblotting and caspase-3 activity.

T84 enterocytes were rinsed with cold phosphate-buffered saline (PBS) and scraped into radioimmunoprecipitation assay (RIPA) lysis buffer supplemented with sodium orthovanadate and protease inhibitor cocktail (Roche, Basel, Switzerland). Samples were normalized for protein concentration using the bicinchoninic acid (BCA) method according to the manufacturer’s instructions (Pierce, Thermo Fisher Scientific). Whole-cell lysates containing 20 μg of total protein were resolved on 10% SDS-polyacrylamide gels and transferred to nitrocellulose membranes (0.45 μm; Bio-Rad). Membranes were blocked with 5% nonfat dry milk (Bio-Rad) in PBS for 1 h and then incubated for 2 h with specific mouse monoclonal primary antibodies against acetylated tubulin (clone 6-11B-1; Sigma) and total tubulin (clone DM1A; Sigma). Immunocomplexes were detected with horseradish peroxidase (HRP)-conjugated goat anti-mouse secondary antibody (catalog no. 31432; Thermo Fisher) followed by enhanced chemiluminescence (GE, Boston, MA). β-Actin was used as an internal reference and probed with an HRP-conjugated mouse monoclonal antibody (clone AC-15; Sigma). Protein bands were visualized using a ChemiDoc-XRS+ apparatus (Bio-Rad), and band intensity was analyzed with ImageJ 1.46r software (National Institutes of Health, Bethesda, MD). For caspase-3 activity, the cells were plated at a density of 1 × 10^6^ per well in 6-well plates and cultured for 24 h at 37°C with 5% CO_2_. Test compounds were applied to the cells and 48 h later, the cells were harvested and caspase-3 activity was measured using a caspase-3 assay kit (Abcam, Cambridge, United Kingdom).

### Statistics.

The analyses were carried out using GraphPad Prism version 9.0 (GraphPad Software, San Diego, CA). Mann-Whitney U test was used for comparing responses between two groups of bacteria, Kruskal-Wallis test followed by Dunn’s posttest was used for bacterial responsiveness to increasing concentrations of exogenous indole and biofilm formation, and one-way analysis of variance (ANOVA) with multiple comparisons was used for determining induction of PXR target genes. A *P* value of ≤0.05 was considered significant.

### Ethics statement.

Studies with bacterial isolate UCH-1 were approved by the Institutional Review Board of Connecticut Children’s Medical Center (no. 16-001). For studies with primary human hepatocytes, the tissue acquisition protocol complied with the regulation issued by the Ethical Committee of the Faculty Hospital Olomouc, Czech Republic and with Transplantation law 285/2002 Coll. Studies with mice were performed in accordance with the Commission for Animal Experiments of the Austrian Ministry of Science (GZBMWFW-66.007/0002-WF/V/3b/2017) and the local ethics committee.

### Data availability.

The sequenced genome of UCH-1 was assembled, annotated, and submitted to the NCBI BioProject database under accession number PRJNA608440 (22).
